# Outcomes of Continuous Positive Airway Pressure in the Management of Patients with Coronavirus (COVID-19) Pneumonia who are not Suitable for Invasive Ventilation

**DOI:** 10.2174/1874306402115010023

**Published:** 2021-06-18

**Authors:** Hnin Aung, Eleni Avraam, Muhammad Ashraf, Nawazish Karim, Sidra Kiran, Muhammed Naeem, Srikumar Mallik, Selva Panchatsharam, George Tsaknis, Raja Reddy

**Affiliations:** 1 Department of Respiratory Medicine, Kettering General Hospital NHS Foundation Trust, Kettering, United Kingdom; 2 Department of Intensive Care Medicine, Kettering General Hospital NHS Foundation Trust, Kettering, United Kingdom

**Keywords:** CPAP, Non-invasive ventilation, COVID-19, Critically ill, Respiratory failure, Ventilation

## Abstract

**Background:**

The optimum management of respiratory failure in patients with coronavirus (COVID-19) infections has been a challenge for physicians across the globe. Many scientific societies have suggested the use of CPAP (continuous positive airway pressure) in severe cases in an effort to reduce invasive ventilation. We investigated mortality outcomes in patients who needed CPAP but were not suitable for invasive ventilation.

**Methods:**

We retrospectively evaluated the mortality outcomes of all consecutive COVID-19 cases with severe type 1 respiratory failure requiring FiO2 >0.6 who were admitted to our hospital between 12th March and 04th May’20. British Thoracic Society guidelines were followed for identifying patients needing CPAP. Their outcomes were recorded and compared with a similar group of patients who had oxygen as a ceiling of care. Prospectively collected data between 5th May and 7th June’20 in similar but smaller groups of patients was also analyzed.

**Results:**

A total of 104 COVID-19 patients with documented Do Not Attempt Resuscitation (DNAR) decision required high fraction of inspired oxygen (FiO2) >0.6(to maintain peripheral oxygen saturation (SpO2)> 92%(SpO2> 88% in COPD patients). Twenty-four patients received CPAP as the ceiling of care, with a mortality rate of 92.5%. The remaining 84 patients who were on oxygen as a ceiling of treatment had 91.7% mortality. Both population groups had a similar number of comorbidities but were less favorable in terms of age in the control group with standard O2 therapy than those who had CPAP support. Overall mortality outcomes from using CPAP therapy did not bring significant mortality benefit (p-value-0.89).

**Conclusion:**

CPAP did not appear to improve the survival of patients with severe respiratory failure due to COVID-19 related pneumonia and were not suitable for invasive ventilation. Further studies are warranted to adequately inform appropriate management strategies for this group of patients.

## 1 BACKGROUND

Severe COVID-19 infection causing respiratory failure requiring high-level care is, unfortunately, an ongoing global problem, posing a considerable strain on hospital resources. There has been a lot of interest in the lung injury physiology of COVID-19 patients. Some expert groups suggest that the Acute Respiratory Distress Syndrome (ARDS) picture caused by COVID-19 behaves differently than other ARDS presentations [1-3]. Additionally, the use of CPAP in ARDS patients has never been proven beneficial in terms of mortality or intubation avoidance to date. The optimum management of such cases with different modalities of respiratory support and their effectiveness in certain groups of COVID-19 patients has not been extensively reported [4]. In England, expert groups and guideline committees have rolled out clear, concise guidance on patient selection regarding escalation of care and eligibility for invasive ventilation. A few studies and strategic proposals highlighted the potential efficacy of CPAP's role in the treatment of Covid-19 [5, 6]. However, the outcomes of specific patients who are not fit for escalation or invasive ventilation but require treatment with CPAP have not been yet reported extensively.

## 2 OBJECTIVES

In our study, we investigated the mortality (in hospital) outcomes of CPAP use in patients with respiratory failure secondary to severe COVID-19 infection who were deemed not fit for invasive ventilation and compared it to patients who were managed on high concentration oxygen alone.

## 3 METHODS

All interventions were carried out at Kettering General Hospital (KGH, United Kingdom), a 600 bedded secondary care hospital serving a population of 330,000. KGH's research ethics committee has confirmed that no ethical approval was required for the study.

### 3.1 Patient and Public Involvement

No patient or public involvement was necessary for this study as it is a service evaluation of nationally published guidelines only.

### 3.2 Patient Selection and Classification

**Fig. (1):**
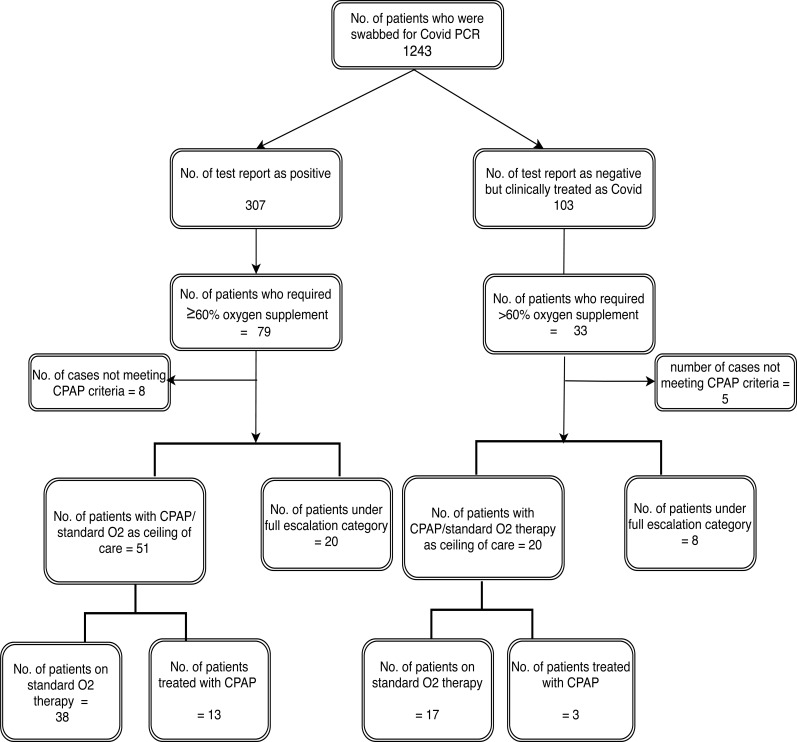
Study subject selection and classification.

We retrospectively investigated all COVID-19 (probable or confirmed) patients with severe respiratory failure who required FiO2> 0.6) and were admitted to our hospital between 15th March and 4th May 2020. All confirmed cases had a positive rRT-PCR nasopharyngeal swab for COVID19. Patients meeting the case definition of ‘probable COVID-19’ as per WHO case definition [7] were also included if they were managed clinically as COVID-19 infection by the treating physician, as false-negative results were common with rRT-PCR testing [8, 9] (Fig. **1**). Patients requiring NIV(non-invasive ventilation) for acute or chronic type 2 respiratory failure due to pre-existing conditions were excluded from the study.

### 3.3 The Decision for Ceiling of the Care Plan and Ventilatory Support

A decision on fitness for invasive ventilation, including DNAR, was recorded in the medical notes at the time of admission after senior clinician review and discussion with the patient as per national guidelines [10], and it does not interfere with the eligibility of CPAP. Continuous positive pressure ventilatory support was considered for patients who met the BTS criteria (patients requiring further ventilatory support despite using 60% O2 concentration/ FiO2>0.6 to maintain SpO2 >92% (88-92% in COPD) for its initiation at the discretion of the attending physician in conjunction with the respiratory team and/or critical care outreach team [11]. Patients who met the criteria for CPAP but were deemed to be too ill to benefit from CPAP due to baseline frailty and poor chance to tolerate ventilatory devices were included in the control group. They received standard oxygen alone.

#### 3.3.1 Inclusion Criteria

- Age 18 and above
- rRT-PCR confirmed or clinically probable COVID-19
- Requiring FiO2 > 0.6 to maintain SpO2 >92% (88-92% in COPD)
- Not suitable for intensive care unit escalation or invasive ventilation with DNAR in place, based on Clinical Frailty Score (CFS) and existing guidelines. Decision recorded on admission, before commen- cement of treatment

#### 3.3.2 Exclusion Criteria

- Fit for Intensive care unit level escalation / invasive ventilation
- Patients requiring BiPAP (Bilevel ventilation) for ‘acute’ or ‘acute on chronic’ type 2 respiratory failure

### 3.4 Statistical Analysis

We retrospectively analyzed the data of all the patients admitted with suspected COVID-19 and analyzed their Vital Signs recorded online. The data were summarized using descriptive statistics, the SPSS system, and results are reported as means and standard deviations, and any differences between the two groups were analyzed using a two-tailed T-test. Cate- gorical variables are summarized numerically, and percentages with any differences analyzed using Chi-squared test.

CFS (Clinical frailty score) and data on comorbidities well known to affect mortality in COVID-19 infection like hyper- tension, diabetes, cardiovascular disease (CVD), cerebrovas- cular accident (CVA), Neutrophil / Lymphocyte ratio (NLR) on admission, and chronic obstructive pulmonary disease (COPD) were collected to compare the two groups (Table **1**).

To improve the validity of the study, whilst analyzing the data for the above two groups, we also prospectively collected data (at arm's length) for any patient who met the same criteria from 5th May to 7th June 2020(Supplementary file). Combined data (retrospective and prospective) was also analyzed to detect any statistically important differences between the groups (Table **2**).

## 4 RESULTS

Between 12th March and 04th May 2020, 71 patients fulfilled the inclusion criteria and were included in the study. Sixteen (16) of them were treated with CPAP, and the rest were treated with oxygen therapy alone. A total of 55 patients were included in the control group who received standard oxygen administration methods ranging from Venturi, Humidified oxygen (delivery through wide-bore nasal cannulae), or non-rebreather reservoir masks. Patients in the CPAP group were treated with either NIPPY 3® ventilator in the CPAP mode or StarMed Ventukit® Up CPAP hoods (Intersurgical SpA, Italy) fitted with viral filters in isolated rooms or cohort bays with close monitoring of observations. Patients were initially commenced on continuous pressure of 1 kPa (kilopascal) with a maximum of 1.5 kPa based on respiratory rate, oxygen saturation, and clinical assessment; arterial blood gases were only taken if clinically indicated.

Study population baseline characteristics are listed in Table **1**. A nearly equal mixture of male and female population numbers is found in their 70s predominantly. Approximately 50% of patients under the category of CPAP therapy have at least 2 comorbidities while 45% of control groups also have at least 2 comorbidities similarly. There was a statistically significant difference between the 2 groups in terms of age and clinical frailty score in favor of the CPAP group. Despite this, there was not any statistical or clinically significant difference in mortality between the two groups. Mortality in the CPAP group was 93.7% (n=16) compared to 92.7% in the control group (n=55).

The prospective arm of the study included a total of 33 patients, of which 8 patients received CPAP, with the rest receiving high flow oxygen only. The mortality in this group was also high, with 91% dying (7 CPAP and 23 in the oxygen group). Mortality remained above 90% when both the retrospective and prospective groups were combined. Similarly, there was no difference in mortality in patients with proven COVID-19 infection and those ‘highly suspected’ cases who were treated clinically as COVID-19 infection. Looking into the duration of hosptial stay, approximately 10 days versus 13 days from admission to the date of discharge, or the mortality outcome in comparison between patients treated with CPAP and the control group.

## 5 DISCUSSION

In our observational study, 15 out of 16 patients with severe COVID-19 infection requiring FiO2 > 0.6 who were deemed unsuitable for invasive ventilation and received CPAP therapy did not survive (93.7% mortality). This was similar to the 92.7% mortality in the control group. Both groups of patients were similar, except for a significant age difference in favor of the CPAP group. With age being a strong predictor of mortality in COVID-19 infection, we would have expected the results to favor the CPAP group. Despite this, a similar percentage of patients survived in this group as in the CPAP group. The high mortality raises doubts about the effectiveness of this modality of treatment in patients who are not suitable for invasive ventilation, even if one were to disregard the oxygen group completely.

During the study period, a further 11 patients with COVID 19 infection required NIV(non-invasive ventilation) for type 2 respiratory failure due to pre-existing respiratory conditions. Most of these patients had a documented DNAR decision on admission. However, their survival rate was 66.7%. Therefore, our study results cannot be generalized to patients requiring NIV for hypercapnic respiratory failure in the context of COVID-19 infection.

Our study, although small, apart from indicating the probable futility of CPAP in patients who are not fit for invasive ventilation, also points to very high mortality in this group of patients who required high flow oxygen (FiO2 > 0.6). However, in the 23 patients deemed suitable for intubation in our hospital (between 12th March and 8th June), CPAP prevented intubation in 13 (56.6%) patients, and overall mortality in this group was 20.8%. It is not very clear as to why the CPAP, which appeared beneficial in patients suitable for full escalation of treatment, didn’t appear to have any clinical benefit in the study patients who are not fit for invasive ventilation.

Our study is certainly not without weaknesses. It is a single-center retrospective study with its attendant biases. However, given the current uncertainty on the optimum management of the severe COVID-19 respiratory failure, as well as the lack of robust data on CPAP in patients who are not fit for ITU escalation, it would be unethical to randomize patients, potentially depriving them of a widely accepted form of treatment. Firstly, one might argue that the level of care for patients who had ‘ward-based CPAP was not ‘on par’ with those who received CPAP in an ITU environment in terms of monitoring and ‘nurse-to-patient ratio, potentially having an impact on outcomes. However, it must be noted that both the patients whose CPAP was managed in the ITU during the initial phase of the pandemic did not survive either. Outside of ITU, the patients were managed in designated areas with expertise in dealing with non-invasive ventilation. The mortality was also similarly high in the prospective group who were treated later on during the pandemic when the breadth of expertise in dealing with CPAP was broader. In our hospital, during the COVID-19 pandemic, we established a 1:3 ‘nurse-to-patient’ ratio for our ‘Level 2’ areas, with continuous monitoring of vital signs and early warning score (EWS), allowing us to maintain high patient safety standards. To our knowledge, in recently published data referring to increased demand of care for COVID-19 patients in all areas, the ‘nurse-to-patient’ ratio has been either similar to ours or even less intense, even in ITU environments due to dilution of staff under the revised COVID recommendations, as well as the inevitable surge [11]. Additionally, one might argue that the patient selection might have been inappropriate, impacting outcomes. To minimize that possibility, we followed all current BTS and Intensive Care Society recommendations for CPAP patient selection and treatment strategies [12]. There were no previous studies that specifically looked at the outcomes of CPAP in patients not suitable for invasive ventilation. A very small retrospective study published recently by Oranger M et al. [13], commented that in 7 such patients, intermittent CPAP improved survival when used early. The limitation of this study is the very small number of participants, and it might be possible that the threshold for commencement of CPAP in UK hospitals might differ from other European settings, as this study recruited patients who required just > 6lts/min oxygen. It is clinically plausible that they might otherwise have survived with the administration of higher concentration oxygen on their own.

## CONCLUSION

Conclusively, in our study, among patients with severe COVID-19 pneumonia who were not candidates for invasive ventilation and treated with CPAP, extremely high mortality is still imminent whether they are placed on CPAP ventilatory support versus high flow O2 alone. These findings were also confirmed in the prospective arm of the study. Presently, RECOVERY RS study is still ongoing in NHS hospitals in the UK to assess the potential mortality benefits of comparing various ventilatory modes among CPAP, HFNO(High flow nasal oxygen) therapy, and standard care arm with oxygen support via face masks. Further studies are warranted to adequately inform appropriate management strategies for this group of patients.

## AUTHOR'S CONTRIBTIONS

Hnin Aung., Eleni Avraam., Z.A., Selva Panchatsharam, Raja Reddy, Muhammed Naeem, Srikumar Mallik, Sidra Kiran, and Nawazish Karim collected and analyzed the data. Raja Reddy and George Tsaknis contributed to the concept of the study, supervised the process. Raja Reddy, George Tsaknis, and Hnin Aung drafted the manuscript.

## Figures and Tables

**Table 1:** Comparison of baseline characteristics and outcomes of retrospective group

	**Retrospective CPAP Group**	**Retrospective** **O2 Group (Control Group)**	**P Value**
Number	16	55	NA
Average Age in years (SD)	72.7 (11.5)	82 (8.19)	0.0006**
Sex	7 (43.7%) male, 9 (56.3%) female	31 (56.4%) male, 24 (43.4%) female	0.26
CFS	4.92	5.5	0.13
COPD	4 (25%)	11 (20.4%)	0.67
Diabetes	4 (25%)	21 (37%)	0 .33
Hypertension	4 (25%)	21 (37%)	0.33
CVA/CVD	8 (50%)	28 (50%)	0 .95
Mean Neutrophil/lymphocyte ratio of >3.3 (SD)	10.97	10.96	0.99
Mortality rate (in Hospital)	93.7%	92.7%	0.89

CPAP = Continuous Positive Airway Pressure, SD = Standard Deviation, CFS = Clinical Frailty Score, COPD = Chronic Obstructive Pulmonary Disease, CVA = Cerebrovascular Accident, CVD = Cardiovascular Disease.

**Table 2:** Detailed analysis results of combined data (retrospective and prospective) groups of patients

	**Combined CPAP Group**	**Combined O2 Group**	**P Value**
Number	24	80	NA
Average Age in years (SD)	74.2 (10.86) Age <70 (8) Age 71-80 (8) Age>80 (8)	82 (7.7) Age <70 (16) Age 71-80 (25) Age>80 (48)	.00006
Sex	9 (37.5%) male, 15 (62.5%) female	40 (50%) male, 40 (50%) female	0.28
CFS	5.0	5.64	0 .064
COPD	33.3%	20.8%	0.207
Diabetes	37.5%	37.6%	0.82
Hypertension	45.8%	40.5%	0.643
CVA/CVD	50%	51.3%	0.913
Mean Neutrophil/lymphocyte ratio (SD)	11.28	10.86	0.87
Mortality rate (in Hospital)	91.7%	92.5%	0.89

## References

[1] Gattinoni L., Chiumello D., Rossi S. (2020). COVID-19 pneumonia: ARDS or not?. Crit. Care.

[2] Marini J.J., Gattinoni L. (2020). Management of COVID-19 Respiratory distress.. JAMA.

[3] Gattinoni L., Coppola S., Cressoni M., Busana M., Rossi S., Chiumello D. (2020). COVID-19 does not lead to a “Typical” acute respiratory distress syndrome.. Am. J. Respir. Crit. Care Med..

[4] Whittle J.S., Pavlov I., Sacchetti A.D., Atwood C., Rosenberg M.S. (2020). Respiratory support for adult patients with COVID-19.. J Am Coll Emerg Physicians Open.

[5] Ashish A, Unsworth A, Martindale J CPAP management of COVID-19 respiratory failure: A first quantitative analysis from an inpatient service evaluation..

[6] Nightingale R, Nwosu N, Kutubudin F, Fletcher T (2020). Is continuous positive airway pressure (CPAP) a new standard of care for type 1 respiratory failure in COVID-19 patients? A retrospective observational study of a dedicated COVID-19 CPAP service.. BMJ Open Respir Res..

[7] https://www.who.int/docs/default-source/coronaviruse/situation-reports/20200423-sitrep-94-covid-19.pdf?sfvrsn=b8304bf0_4.

[8] Woloshin S, Patel N,S., Kesselheim A (2020). False Negative Tests for SARS-CoV-2 Infection — Challenges and Implications.. Perspective..

[9] Arevalo-Rodriguez I, Buitrago-Garcia D, Simancas-Racines D False-negative results of initial rt-pcr assays for covid-19: A systematic review.. COVID-19 SARS-CoV-2 preprints from medRxiv and bioRxiv..

[10] https://www.gmc-uk.org/ethical-guidance/ethical-guidance-for-doctors/treatment-and-care-towards-the-end-of-life/cardiopulmonary-resuscitation-cpr.

[11] Litton E., Bucci T., Chavan S., Ho Y.Y., Holley A., Howard G., Huckson S., Kwong P., Millar J., Nguyen N., Secombe P., Ziegenfuss M., Pilcher D. (2020). Surge capacity of intensive care units in case of acute increase in demand caused by COVID-19 in Australia.. Med. J. Aust..

[12] (2020). https://www.brit-thoracic.org.uk/about-us/covid-19-information-for-the-respiratorycommunity/Guidance.

[13] Oranger M., Gonzalez-Bermejo J., Dacosta-Noble P., Llontop C., Guerder A., Trosini-Desert V., Faure M., Raux M., Decavele M., Demoule A., Morélot-Panzini C., Similowski T. (2020). Continuous positive airway pressure to avoid intubation in SARS-CoV-2 pneumonia: A two-period retrospective case-control study.. Eur. Respir. J..

